# Mental Health and (Online) Behaviors during the COVID-19 Pandemic in Spain: A Network-Based Approach

**DOI:** 10.3390/bs14090735

**Published:** 2024-08-23

**Authors:** Maribel Serrano-Macias, Javier Alvarez-Galvez

**Affiliations:** 1 Department of General Economy (Sociology), Faculty of Nursing and Physiotherapy, University of Cádiz, 11009 Cádiz, Spain; isabel.serrano@uca.es; 2 Computational Social Science DataLab (CS2 DataLab), University Institute for Social Sustainable Development, University of Cádiz, 11405 Jerez de la Frontera, Spain

**Keywords:** mental health, health behaviors, lifestyles, social network analysis, mixed graphical models

## Abstract

The COVID-19 pandemic has contributed to the increase in mortality and morbidity rates globally, but it has also led to a generalized worsening of mental health and risk behaviors in different population groups regardless of the measures adopted by different governments. In this paper, using data from a Spanish survey of emotional well-being, we aim to explore through mixed graphical models the complex structure of relationships between the mental health of populations, their lifestyles, and forms of cultural and leisure consumption during the pandemic. The results bring to light some interesting findings, such as the association between teleworking and greater rest or greater stress with the use of social media, a variable that enables the connection with other mental health problems of greater severity. Increased physical activity and the consumption of streaming content at home, as well as increased care for family, friends, and neighbors, are some of the variables that show relevant associations. These findings highlight the usefulness and versatility of this network approach for the study of health behaviors and health outcomes, which offer the researcher a holistic and organic view of the relational structure of complex data characterized by high dimensionality and variables with different levels of measurement.

## 1. Introduction

The COVID-19 pandemic has contributed to the increase in mortality and morbidity rates globally [1], but it has also resulted in the generalized worsening of mental health and risk behaviors in different population groups regardless of the measures adopted by different governments [2]. The pandemic has also disrupted access to mental health care, as many services have had to be delivered remotely or have been scaled back due to staffing and medical resource limitations [3]. This contraction in mental health services has made it more difficult for people to receive the support they need [4]. In addition, the fear and uncertainty linked to the new global situation [5], as well as the governmental implementation of containment measures such as social distancing and lockdowns, have widely contributed to an increase in mental health problems such as stress, anxiety, depression, and substance abuse [6], particularly among the most vulnerable groups (children, young adults, migrants and ethnic minorities, older population, people with disabilities, and, in general, low-income population) [7,8].

In this context of global emergency, the COVID-19 pandemic has had a broad impact on human behavior. Trying to control the spread of the SARS-Cov-2 virus, the pandemic has brought radical changes in our daily routines and the way we normally interact with others [9]. The travel limits and the closure of countries’ borders have significantly reduced both local and global mobility. The economic slowdown resulted in very sharp declines in mobility, which were even more pronounced among groups with lower socioeconomic status. [10]. Mobility patterns were also found to be closely linked to existing social inequalities [10]. On the other hand, in terms of social relations, the restrictions of large social meetings and the governmental recommendations to maintain physical distance from others led to a decline in in-person socializing, while virtual meetings exponentially grew during this period (including online gatherings with family members, friends, and work colleagues) [11].

At the occupational level, many people have had to work from home or have even lost their jobs due to the new containment measures and subsequent closure of leisure services and shopping centers [12]. Specifically, the closure of non-essential stores and businesses and the shift towards online shopping have changed the way that people buy and consume [13]. In fact, the new restrictions during this period have incorporated forms of consumption oriented towards the home and the family context (including consumption of food and leisure products, as well as improvements in housing) [14]. The pandemic has changed the way that we work and interact with our co-workers and is even leading to new changes in our workday, which in many cases is progressively becoming more flexible [15,16].

The pandemic also affected the educational sphere of our societies. For instance, the closure of educative institutions (i.e., schools, universities, and other academies) has accelerated the introduction of online teaching, even in those institutions based on face-to-face education, changing the way that people learn and interact with their classmates and teachers [17,18]. Many students have had to adapt to online education, and children have had to be increasingly supported in the use of technology by parents who sometimes have neither the technical means nor the adequate skills for online teaching [19].

Consequently, the COVID-19 pandemic has profoundly disrupted many aspects of our daily lives that, directly or indirectly, have affected the basic social structures of our societies (family, communities, forms of work, social classes, economy and forms of consumption, and even educational and health institutions). As in other moments of social and economic crisis, this hasty process of social change has generated important changes in opinions, attitudes, and (health) behaviors due to the need to adapt to the new circumstances. However, behavioral change strategies have also been used to fight the pandemic [20].

While an increasing number of studies are showing the impact of the pandemic on mental health, attitudes, and health behaviors, to date there is no clear picture of the interrelationship between the different conducts (whether pro- or anti-social) that have taken place during this period of global uncertainty and the mental health of the population [2,21]. Evidence from a recent multi-country study showed that prosocial behavior was associated with higher wellbeing in all regions. In terms of predictors, high levels of perceived social support were most strongly associated with prosocial behavior, followed by high levels of perceived stress, positive affect, and psychological flexibility (greater adaptive capacity to change) [22]. That is, although we have observed both positive and negative attitudes and behaviors during the pandemic [23], further work is needed to identify specific behaviors, lifestyles, and sociocultural practices that could increase the mental resilience of these groups in the face of future pandemics.

In this paper, using data from the emotional well-being survey of the Sociological Research Center (Centro de investigaciones Sociológicas, CIS), we aim to explore through mixed graphical models and social network analysis the complex structure of relationships between the mental health of the Spanish population, their lifestyles, and forms of cultural and leisure consumption during the first months of the COVID-19 pandemic.

## 2. Materials and Methods

The present study is characterized by a descriptive cross-sectional design, which aimed to identify—by implementing mixed graphical models (MGM)—the latent structure of interrelationships between co-occurring feelings and changes in daily activities that configured the complex patterns of the health behaviors and lifestyles of the Spanish population during the confinement and lockdown period at the beginning of the new health crisis.

### 2.1. Data and Variables

This study is based on data from the study on Emotional Wellbeing during the COVID-19 pandemic (study number 3285) of the Sociological Research Center (CIS). The CIS is an independent entity assigned to the Ministry of the Presidency. The main remit of the CIS is to contribute scientific knowledge on Spanish society. The CIS collects the necessary data for research in very different fields, from trends in public opinion to applied research. The study was carried out from 5 to 8 June 2020, using a computer-assisted telephone interview system (CATI), with interviews being about 15 min long. The survey was national in scope and the universe comprised the Spanish population of both sexes aged 18 and over. The final sample size was 937 interviews carried out by proportional allocation. The information used in the calculation of the weighting coefficients was, on the one hand, the distribution of the Spanish population aged 18 years old and over according to Autonomous Communities and cities. A total of 488 municipalities and 50 provinces were considered among the sampling points.

The random selection of landline and mobile phones was carried out with a percentage of 30.9% and 69.1%, respectively. Individuals were selected by applying sex and age quotas. The strata were formed by crossing the 17 autonomous communities and the two autonomous cities with the size of habitat, divided into 7 categories: less than or equal to 2000 inhabitants; from 2001 to 10,000; from 10,001 to 50,000; from 50,001 to 100,000; from 100,001 to 400,000; from 400,001 to 1,000,000; and more than 1,000,000 inhabitants. The questionnaires were administered by computer-assisted telephone interview (CATI). A sampling error was considered for a confidence level of 95.5% (two sigma) and P = Q; the actual error is ±3.3% for the whole sample, assuming simple random sampling.

The mental variables selected for this study were the following: (1) felt unhappy; (2) felt depressed; (3) felt uneasy; (4) felt no enjoy; (5) felt no energy; (6) felt alone; (7) felt tired; (8) felt sad; (9) felt worried; (10) felt stressed. They fell within the following categories: (1) All or most of the time; (2) A good part of the time; (3) Some of the time; and (4) At no time or almost no time. The behavioral indicators, encoded as binary indicators where 0 was No and 1 Yes, were (11) during this period has changed your diet; (12) your physical activity; (13) your health; (14) your relationship with your family; (15) your relationship with your neighbors; (16) your involvement in volunteer and community outreach activities; (17) your leisure activities; (18) your friendships, social relationships; (19) your work/studies/main activity; (20) used more board games with family, partner or roommates; (21) you have connected more by video call with family or friends; (22) you have connected by videoconference more with your work or study colleagues, teachers, bosses, etc.; (23) exchanged more messages, photos, videos, jokes in their chat groups; (24) watched more series, movies, documentaries, sports events, etc. on television; (25) read more books and magazines; (26) have been more aware of neighborhood and/or volunteer activities; (27) followed the news in the media more often; (28) you have been more aware of your social networks; (29) have done more online shopping; (30) have done more sports activities at home; (31) you have dedicated more time to household tasks, cooking, tidying up, gardening, etc.; (32) has been more attentive to family members (contacting more by phone with parent(s), supervising the family, etc.); (33) you have rested and slept more; (34) has more teleworking. We also included the sex of the respondent to study differences by these subgroups.

As we were working with secondary data from a national public survey, it was not necessary to go through an ethics committee; in any case, the interviews were conducted with informed consent and the study has been approved by the CIS ethics committee.

### 2.2. Statistical Analysis

In this study, the *mgm* package [24] was applied to estimate a k-order mixed graphical model (MGM) of behavioral patterns based on pairwise associations. This library estimated MGMs using a ‘nodewise’ estimation method with a penalization based on least absolute shrinkage and selection operator regularization (LASSO) [25]. Although the graphic representation of associations tends to make it easier to obtain a first impression of a data structure, the interpretation of these visual models can still be complex enough when the resulting networks have a high density of vertices (i.e., nodes) and edges (i.e., links or connections) [24]. Therefore, with the objective of providing an easy interpretation of the results by reducing non-significant association, in MGM the GLASSO algorithm (i.e., Graphical LASSO) is a frequently used method to produce ultimately sparser networks with a lower density [26]. The GLASSO algorithm forces low partial correlation coefficients to tend to zero, which favors the sparsity and general visualization of the networks.

Consequently, the global density of the graph can be controlled by various fitting parameters that can be modified to produce easier to read and interpretable solutions. The estimation algorithm needs us to make an assumption about the highest order interaction between predictors in the graph. The algorithm includes an L1 penalization to obtain a sparse estimate that is controlled by the regularization parameter lambda (λ), using cross-validation or the extended Bayesian information criterion (EBIC) [24].

In our case, EBIC was used to select an optimal fit of the fitting parameter so that the strongest relationships were retained in the graph (i.e., maximizing true positives), since this method is more conservative than the cross-validation. The EBIC itself has a setting parameter gamma (γ), which controls the balance between sensitivity and precision, based in a default value of 0.25 (the lower the γ hyperparameter, the higher the density of the graph) [27]. Finally, the LASSO method produces a parsimonious network in which small edges are shrunk to zero, hence only the most robust and consistent associations are presented (i.e., using edges or links between nodes), while non-statistically significant relationships are excluded so the likelihood of false positives is reduced [28].

Given the diversity of behavioral and mental health indicators used in this study and the inherent complex structure of association patterns identified in the literature, some relations were expected to be non-relevant and therefore more likely to be contracted to zero through this method. To avoid the loss of these associations, the γ value would also be set to 0 (which turns EBIC into BIC), as well as to other higher values that would allow us to identify the correct association patterns while losing relevant information.

In a subsequent stage, the resulting network model was visually represented using the *qgraph* package [29]. The resulting graph was an undirected weighted network showing the pairwise relationships between the different variables in the model, with an undirected graph *G* = (*V*, *E*) consisting of a collection of nodes or vertices *V* = {1,2,…,…,*p*} and a collection of edges *E ⊂ V × V*. In these models, the graphical model G was then taken from the parameter estimates. Thus, the resulting MGM consists of a set of elements or variables (i.e., mental health and behavioral indicators mentioned in the previous section), represented by circles, and a set of links depicting the relationships between these variables [30,31]. The thickness of these connectors represents the strength of the associations between the variables included in the model; conversely, the absence of a link means that there are no relationships or that they are too weak to be statistically relevant. In the MGM, these connections capture partial correlations, i.e., the correlation between pairs of variables when controlling for all other relationships in the dataset, which has the advantage of avoiding spurious correlations.

After this step, the resulting network structure was characterized through three main centrality measures: (1) degree (measures the number of connections between variables of interest); (2) closeness centrality (this measure quantifies the number of indirect connections a node has with other nodes); and (3) betweenness centrality, which describes how important a node (i.e., variable in the model) is in the average path between two other nodes or variables [32]. In general terms, this set of centrality metrics would provide us with information on the most relevant (or centrally positioned) variables within the overall relational structure.

Lastly, between-node predictability was estimated for all the variables included in the network model, and the proportion of correct classifications was evaluated by measures of accuracy (Acc) and normalized accuracy (nAcc). Bootstrapping methods were used to assess the accuracy of the estimated network. The resample function implemented in the MGM was used, and to analyze the robustness of our network obtained using γ = 0, the same estimation procedure was repeated with the default values of γ = 0.25 and γ = 0.50; estimations of the three networks were compared.

## 3. Results

**Figure 1:**
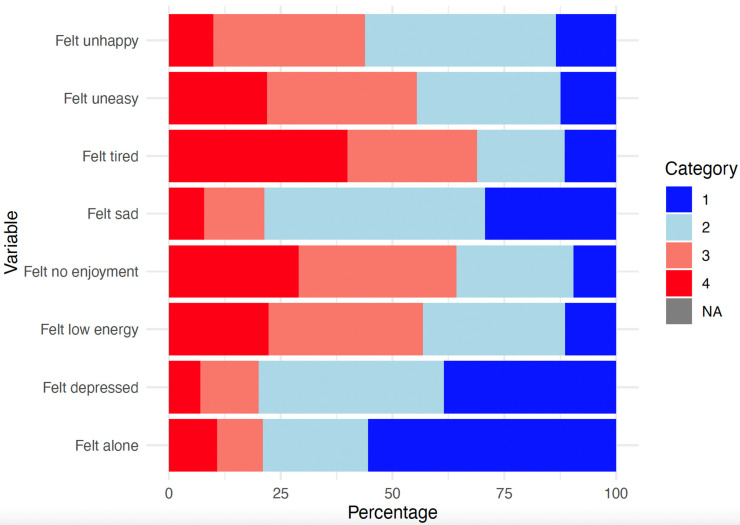
Distribution of responses in the mental health variables (%). The response categories are (1) All or most of the time; (2) A good part of the time; (3) Some of the time; and (4) At no time or almost no time.

**Figure 2:**
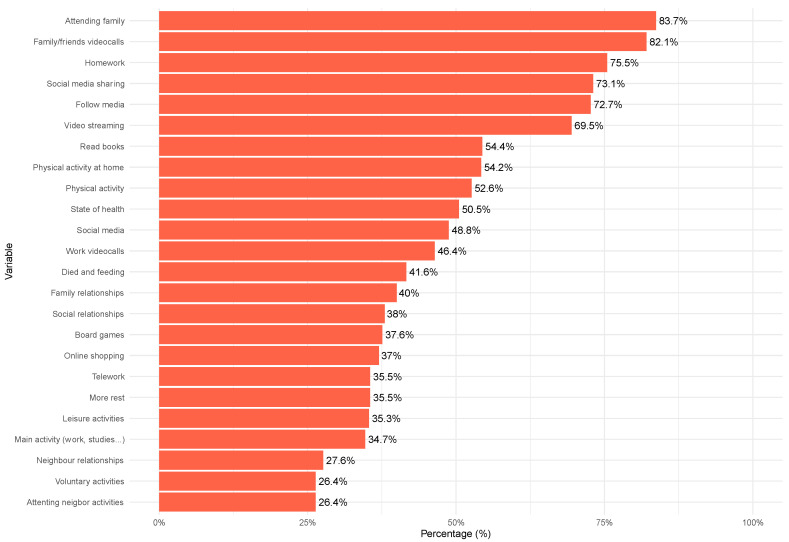
Distribution of responses in behavioral variables (%).

Table 1 shows descriptive statistics for the variables included in the model. The responses for these variables are based on a total sample of 937 cases, with no missing values. Percentages for mental health-related variables are described in Figure 1 and behavioral indicators in Figure 2.

Figure 1 displays the distribution of responses for mental health indicators among the study participants. Each bar represents the percentage distribution of responses across four Likert scale categories for the following variables: feeling alone, feeling depressed, feeling low energy, feeling no enjoyment, feeling sad, feeling tired, feeling uneasy, and feeling unhappy. The data reveal that a notable proportion of participants reported higher levels of negative emotional states (e.g., low energy, tiredness, lack of enjoyment, and generally greater unhappiness.), highlighting the widespread prevalence of mental health concerns within the study population.

Figure 2 presents the percentage of participants engaged in the activities and behaviors measured. The highest participation rates are seen in attending neighbor activities (83.7%) and voluntary activities (82.1%), followed by strong engagement in neighbor relationships (75.5%), main activities such as work or studies (73.1%), and leisure activities (72.7%). Notable engagement is also reported in family relationships (48.8%), social media usage (41.6%), and physical activity (38%). Activities with lower percentages include family/friends videocalls (27.6%) and attending family (26.4%). These results highlight the diverse range of behaviors participants are involved in, with a significant emphasis on community and social interactions.

**Figure 3:**
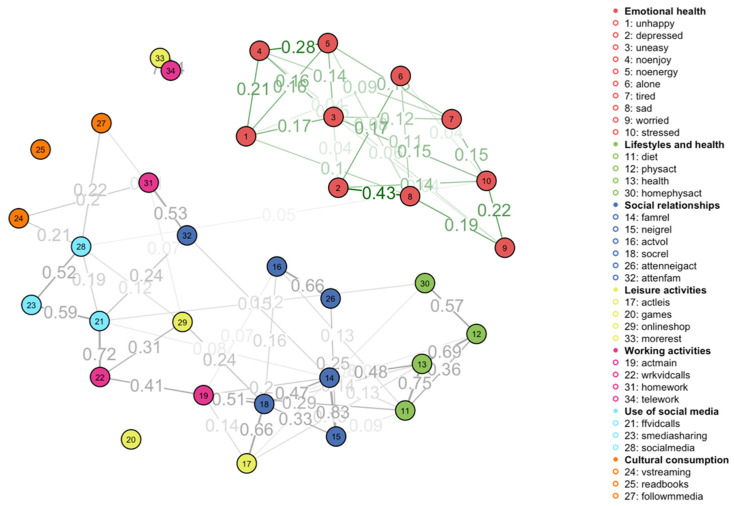
Mixed graphical model using the full dataset (k  =  2).

The visual results of the MGM are described below (Figure 3), showing the network structure of relationships between the different predictors of mental health and associated behaviors during the pandemic period that were used in this analysis. In this network, each node represents a specific variable in the analysis, categorized into different groups: (a) Mental health; (b) Lifestyles and health; (c) Social relationships; (d) Leisure activities; (e) Working activities; (f) Use of social media; and (g) Cultural consumption. The edges between nodes indicate the relationships between these variables, with the thickness of the edges representing the strength of these associations.

### 3.1. Mental Health, Lifestyles, and Social Relationships

At a global level, we can observe a clear separation between the mental health indicators and those referring to changes in the behavior of the Spanish population during the pandemic period. There is a significant interrelationship between all the measures of mental health. Feelings of depression and sadness are the most strongly related, along with feelings of lack of enjoyment, energy, and general unhappiness.

While the relationships between the mental health metrics are less striking, the changes in behaviors reveal some sociologically relevant interrelationships. For example, among lifestyles, there is a strong association between physical activity at home and outdoors physical activity, showing that people who did physical activities and sport before the pandemic tried to also maintain these practices during the period of confinement and lockdown. It also shows the interconnection of these activities with the person’s perception of health (which would normally be more positive among these groups) and healthy eating habits.

Interestingly, it is the self-perception of health that is directly linked to changes in family relationships, which is a predictor that articulates the whole block of indicators referring to changes in social and personal relationships. Indeed, it is changes in family relationships that explain changes in relationships with other groups, as well as the attention paid both to family members and neighbors. Changes in family relationships also explain the increase in video calls with family members with whom direct contact was no longer possible due to the confinement itself. At the same time, the increase in care for family members has led to an increase in household tasks (helping with dependents, supporting the education of children at home, and carrying out household activities).

### 3.2. Leisure and (Tele)Working Activities

Leisure activities present more diffuse connections in the overall behavioral network structure. The performance of leisure activities is linked to changes in relationships in general, although the relationships with self-perceived health and changes in the main activity (i.e., work or studies) are also significant. Online shopping is associated with the use of social media, increased housework activities and teleworking. This shows that the increased exposure to online media in daily activities, both at home and at work, is clearly associated with current forms of online commerce and consumption. On the other hand, the new forms of teleworking are strongly associated with increased rest for the individuals interviewed, which explains the perfect accuracy in these indicators (Table 2). These findings are relevant from the point of view of the relationship between professional activity and quality of life. Finally, the practice of board games remains as an isolated practice with respect to the rest of the observed behaviors, indicating that this type of practice is weakly associated with new forms of leisure linked to the use of new social media platforms, as well as to new forms of online commerce and social relationships through new social media platforms. In any case, we should bear in mind that the absence of a relationship with this particular variable may also be related to the particular situations of each family nucleus (the size of the family or the time available for the group of members).

### 3.3. Social Media Use and Cultural Consumption

The three variables referring to the use of social media platforms (making online video calls, social media use, and media sharing through apps and online chats) are articulated as a triad in the context of the behavioral network. In relation to other predictor communities, it is observed that the increase in making video calls is linked to the increase in attention to family members, which shows that, during confinement, new media have been a way of providing attention to the family and being closely connected. In the network, the most relevant finding is the connection between the use of social media and the stress experienced, which demonstrate the core role of these platforms in the explanation of mental health and mental health conditions. Specifically, social media use variables like media sharing (i.e., messages, images, and videos) through online chats (23), social media use (28), and following the news in the media more often (27) form a distinct group within the graph, with social media use being the connecting factor between mental health and other behaviors. Therefore, social media use is particularly central, connecting with several other nodes, indicating its importance in the network.

When we observe cultural consumption practices, we can again appreciate the interconnection between the use of social media and media monitoring, as well as the consumption of movies, series, or documentaries. This interconnection evidences the central role that new social media platforms (such as, for example, Twitter, Facebook, Instagram or TikTok) play in the media information and audio–visual materials that we end up consuming. Finally, as was the case with the use of board games, book reading remains a social practice detached from the set of practices inherent to the online and social media environment.

Therefore, this network underscores the complex interplay between behaviors and mental health during the COVID-19 pandemic, with social media use emerging as a crucial intermediary connecting mental health variables to broader lifestyle and behavioral factors.

Table 2 describes the Root Mean Squared Error (RMSE) and the proportion of explained variance (R2)for continuous variables, and the proportion of correct classification or accuracy (CC) and the proportion of correct classification normalized by the marginal distribution of the variable at hand (nCC) for categorical indicators.

**Figure 4:**
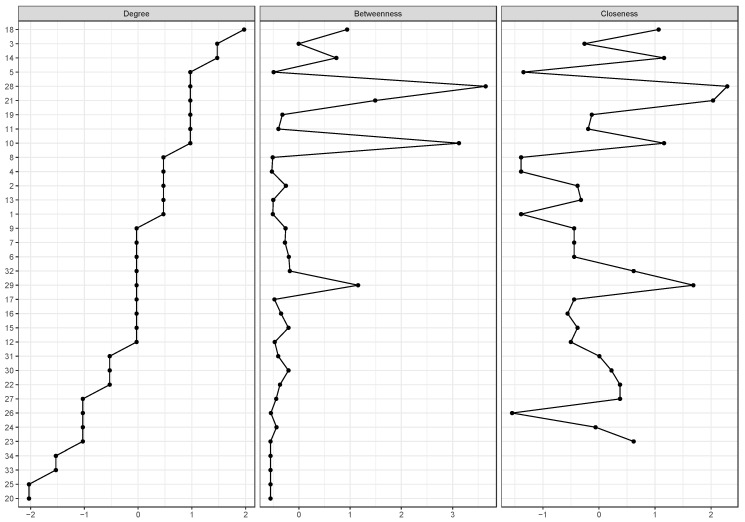
Centrality measures for variables in the model.

Figure 4 shows three centrality measures that were used to describe the general relevance of the different variables in the network model: (a) degree; (b) betweenness; and (c) closeness centrality. The degree measure describes the number of connections between variables of interest, the closeness centrality index quantifies indirect connections a node has with other nodes, and the betweenness centrality measures how important a node (i.e., a variable in this case) is in the average path between two other nodes (variables). Although based on the degree we observe that the indicators on family relationships are those that present the greatest centrality in the set of behaviors, the measure of betweenness and closeness centrality shows the articulating capacity of the use of social media (and its connection with stress) between behavioral changes and the mental health of the population in Spain. Likewise, variables related to making video calls with friends and family, together with online shopping, also acquire a central role in the set of relationships.

The indicator with a higher betweenness centrality is social media use (28), which again indicates the relevance of this variable in connecting the different variables of communities in the network. In other words, this means that social media use serves as a bridge between different variable clusters, including mental health and other activities. Specifically, social media use (28) connects with variables such as online shopping (29) and physical activities (12), indicating that social media usage could mediate the impact of these behaviors on mental health.

In relation to closeness centrality (i.e., that which indicates the most central nodes in the network as a whole), we observed that making video calls with family and friends (21) is the most important in the group of variables of behaviors and activities performed during the first months of the pandemic, closely followed by online shopping (29), which also played a central role in our lives during the period of confinement. These results highlight the importance that personal relationships such as online shopping had during this period.

Finally, two models were generated based on gender and two for youth and adults; however, the results showed no statistically significant differences based on these socio-demographic factors.

## 4. Discussion

In this article, we have explored the complex structure of relationships between the mental health of the Spanish population and their behaviors during the period of confinement in the COVID-19 pandemic. Unlike previous work based on the study of specific behaviors during the recent health crisis, this study offers a relational approach that allows us to explore and understand the association between mental health and the behaviors that the population has modified g in an adaptive way during this period of pandemic, including eating habits, forms of leisure, sports practices, social and personal relationships, and even forms of cultural consumption. In particular, our findings highlight the critical role of social media and new online platforms (including those for media sharing and online shopping) during the COVID-19 pandemic in connecting people (family and friend), work, daily activities (diets and physical activity), and emotions.

As previous studies have demonstrated [33], the emotions experienced highlight the significant relationship between feelings of depression, sadness, lack of enjoyment, and energy, feelings that were articulated around experiences of loneliness and unhappiness (i.e., the variables that had the most central role within the relationship structure). Although in our study we have not detected significant differences in terms of age and sex, recent studies have shown an increase in feelings of loneliness, especially in older age groups, i.e., those who have suffered a greater loss of social support due to a lack of digital literacy [34,35,36]. Thus, while young people have been able to stay relatively connected through new social networking and media sharing platforms, older people have seen their family and neighborhood relationships considerably limited, which has contributed to an increase in the cognitive deterioration of people in vulnerable and dependent situations [37].

Among the behaviors, practices of an ambivalent nature are observed. On the one hand, there are behaviors that could be described as “pro-social” [22], such as a greater frequentation of online relationships and attention to family, friends, and neighbors, which shows the resilience of communities to cope during periods of health crises such as the COVID-19 pandemic. There is also evidence of positive aspects in the adaptation of the population to physical exercise and online forms of work [38], which, as shown, have contributed to greater rest and family reconciliation for workers (through family activities: board games, household chores, among others). On the other hand, there is evidence of an increase in screen time activities, such as increased use of social networking platforms, greater consumption of news and media, video streaming platforms, teleworking, video calls, and online shopping, while cultural practices such as reading books and playing family games take a back seat and are generally disconnected from online behaviors. Recent studies also show increased screen exposure time during the period of confinement, especially among younger groups, although, as in our study, no statistically significant differences between women and men have been detected [38,39].

However, the link between social media use and stress during this period of health crisis is noticeable due to the central role of this practice in the global structure of the network. Indeed, several studies point to the harmful effects of social media on mental health, particularly among young people, who are the most exposed to these new platforms [40,41,42,43]. While the relationship between (passive) social media use and the emotions of stress, anxiety, and depression have been identified in the literature, it is striking that among a broad set of behaviors in our model that changed during the pandemic period, it is the use of these social media platforms that articulates the relationship with the mental health component. These findings show that although these new technologies have made it possible for us to connect with family, friends, and colleagues, they have also generated fatigue and weariness among the population due to their own inability to deal with the incessant flow of information. Indeed, recent studies show that, in the context of COVID-19 infodemics, increased media exposure can have a negative impact on mental health outcomes [44,45].

Finally, it is necessary to mention some limitations. First, when interpreting models that describe a complex and broad structure of predictors, it is necessary to contextualize these relationships in order to adequately characterize the phenomenon to be described. Second, it is necessary to indicate that the cross-sectional nature of the study does not prevent us from drawing conclusions about the possible causal relationships that could define the structure of behaviors and emotions experienced by the population under study. Thirdly, we have to mention that the results did not show statistically significant differences based on gender and age, which is related to the fact that we worked with a relatively small sample for the large set of variables analyzed. Additionally, it is worth mentioning that the absence of differences according to sex and age groups could be due to the smaller size of the samples once the different subpopulations were disaggregated. However, we suppose that taking into account the exceptionality of the pandemic context suffered as a global shared experience that was retransmitted univocally through mass media and social media, we think that the media filter may have contributed to the homogenization of behaviors and the mental situation of the population. In any case, given the absence of data to clarify our new research questions, these are assumptions that would need to be thoroughly addressed through qualitative and longitudinal studies that can shed light on these aspects in the near future.

As an advance of our work, we must also emphasize that, in contrast to other studies that characterize relationships between behavioral and mental health variables in static models that generally describe small sets of indicators, the network model generated by MGM offers us an organic and relational view of the behaviors and emotions experienced by the Spanish population. Therefore, the usefulness of this type of method for the characterization of complex sociological phenomena that articulate different constructs and units of measurement becomes evident.

## 5. Conclusions

As this paper shows, the COVID-19 pandemic has had a significant impact on human behavior and mental health. From a sociological perspective, it is evident that the pandemic has generated changes in social structures, the norms that govern our ways of life, our daily routines, and also in the patterns of social interaction, in short, changes that have arisen both spontaneously and others that have been imposed by government authorities.

The pandemic has also had a significant impact on our mental health. The feelings of fear, uncertainty, and isolation that many people experienced during the pandemic have led to increased levels of loneliness, stress, anxiety, unhappiness, and depression. Changes in social norms and daily routines have also contributed to feelings of disorientation and loss of control, which consequently may have had negative effects on mental health. This impact has been particularly pronounced among vulnerable populations, such as those who have lost their jobs, those living in poverty and at risk of exclusion, and those who lacked social support or live in a situation of dependency (elderly, people with disabilities, among others). However, it is necessary to consider that it is still too early to assess the actual impact of the pandemic, as health conditions such as depression or the negative impact of risk behaviors (such as increased alcohol and drug use) do not always appear immediately.

In this sense, it is necessary to work on prevention among particularly vulnerable groups, such as the elderly, who may experience a lack of social support and loneliness, or even the young people of our societies who are immersed in a tidal wave of information that they cannot always process.

## Figures and Tables

**Table 1:** Descriptive statistics.

ID	Variable	Min	Mean	SD	Max
1	Felt unhappy	1	2.040	0.865	4
2	Felt depressed	1	1.523	0.742	4
3	Felt uneasy	1	2.201	0.993	4
4	Felt no enjoy	1	2.372	1.050	4
5	Felt without energy	1	2.230	0.999	4
6	Felt alone	1	1.354	0.745	4
7	Felt tired	1	2.412	1.160	4
8	Felt sad	1	1.643	0.770	4
9	Felt worried	1	2.190	0.904	4
10	Felt stressed	1	1.780	0.906	1
11	Died and feeding	0	0.416	0.493	1
12	Physical activity	0	0.526	0.500	1
13	State of health	0	0.505	0.500	1
14	Family relationships	0	0.400	0.490	1
15	Neighbor relationships	0	0.276	0.447	1
16	Voluntary activities	0	0.264	0.441	1
17	Leisure activities	0	0.353	0.478	1
18	Social relationships	0	0.380	0.486	1
19	Main activity (work, studies, etc.)	0	0.347	0.476	1
20	Board games	0	0.376	0.485	1
21	Family and friends videocalls	0	0.821	0.384	1
22	Work videocalls	0	0.464	0.499	1
23	Media sharing (message, image, videos)	0	0.731	0.444	1
24	Video streaming	0	0.695	0.461	1
25	Read books	0	0.544	0.498	1
26	Aware of neighbor activities	0	0.264	0.441	1
27	Follow media (news)	0	0.727	0.446	1
28	Social media use	0	0.488	0.500	1
29	Online shopping	0	0.370	0.483	1
30	Physical activity at home	0	0.542	0.498	1
31	Homework	0	0.755	0.431	1
32	Attention family	0	0.837	0.370	1
33	More rest	0	0.355	0.479	1
34	Telework	0	0.355	0.479	1

**Table 2:** MGM classification results.

ID	Variable	RMSE	R^2^
1	Felt unhappy	0.772	0.404
2	Felt depressed	0.705	0.503
3	Felt uneasy	0.754	0.431
4	Felt no enjoy	0.747	0.442
5	Felt without energy	0.736	0.457
6	Felt alone	0.880	0.225
7	Felt tired	0.843	0.289
8	Felt sad	0.699	0.511
9	Felt worried	0.835	0.302
10	Felt stressed	0.782	0.388
**ID**	**Variable**	**CC**	**nCC**
11	Died and feeding	0.780	0.472
12	Physical activity	0.796	0.570
13	State of health	0.822	0.640
14	Family relationships	0.815	0.539
15	Neighbor relationships	0.795	0.259
16	Voluntary activities	0.790	0.202
17	Leisure activities	0.758	0.314
18	Social relationships	0.805	0.486
19	Main activity (work, studies, etc.)	0.772	0.342
20	Board games	0.624	0.000
21	Family and friends videocalls	0.859	0.214
22	Work videocalls	0.702	0.359
23	Media sharing	0.776	0.167
24	Video streaming	0.695	0.000
25	Read books	0.544	0.000
26	Aware of neighbor activities	0.778	0.158
27	Follow media (news)	0.727	0.000
28	Social media use	0.702	0.389
29	Online shopping	0.699	0.187
30	Physical activity at home	0.707	0.359
31	Homework	0.780	0.104
32	Attention family	0.837	0.000
33	More rest	1.000	1.000
34	Telework	1.000	1.000

## Data Availability

Data for this publication are available at https://www.cis.es/detalle-ficha-estudio?origen=estudio&idEstudio=14512.
